# The Nuffield Early Language Intervention (NELI) programme is associated with lasting improvements in children's language and reading skills

**DOI:** 10.1111/jcpp.14157

**Published:** 2025-03-12

**Authors:** Charles Hulme, Gillian West, Mariela Rios Diaz, Sarah Hearne, Caroline Korell, Mihaela Duta, Margaret J. Snowling

**Affiliations:** ^1^ University of Oxford Oxford UK; ^2^ Oxford Brookes University Oxford UK; ^3^ OxEd and Assessment Ltd Oxford UK; ^4^ University College London London UK

**Keywords:** Language intervention, randomised controlled trial (RCT), follow‐up study, oral language, reading skills

## Abstract

**Background:**

Oral language skills are a critical foundation for education and psychosocial development. Learning to read, in particular, depends heavily on oral language skills. The Nuffield Early Language Intervention (NELI) has been shown to improve the language of 4–5‐year‐old children entering school with language weaknesses in four robust trials. To date, however, there is limited evidence on the durability of the gains produced by the intervention, and some have argued that the effects of such educational interventions typically fade‐out quite rapidly.

**Methods:**

A large‐scale effectiveness trial of the NELI intervention implemented under real‐world conditions produced educationally meaningful improvements in children's language and reading abilities. Here, we report follow‐up testing of children from this study conducted approximately 2 years after the completion of the intervention.

**Results:**

At 2‐year follow‐up, children who had received NELI had better oral language (*d* = 0.22 or *d* = 0.33 for children with lower language ability), reading comprehension (*d* = 0.16 or *d* = 0.24 for children with lower language ability) and single‐word reading skills (*d* = 0.16 or *d* = 0.22 for children with lower language ability) than the control group.

**Conclusions:**

Our data show that, although fade‐out effects are common in educational research, a widely used language intervention produces durable improvements in language and reading skills, with educationally important effect sizes. These findings have important theoretical and practical implications.

## Introduction

Language skills are a critical foundation for education and psychosocial development and are particularly closely related to literacy (Hjetland, Brinchmann, Scherer, Hulme, & Melby‐Lervag, 2020; Hulme, Nash, Gooch, Lervag, & Snowling, 2015; Snow, 2016) and numeracy development (Chow & Ekholm, 2019; Hornburg, Schmitt, & Purpura, 2018). Children from disadvantaged homes typically show poorer language development than children from more privileged backgrounds (Guo & Harris, 2000; Hart & Risley, 1995; Roulstone, Law, Rush, Clegg, & Peters, 2011; Sampson, Sharkey, & Raudenbush, 2008; Sirin, 2005). It follows that interventions to improve language skills are potentially a powerful way to reduce educational and occupational inequalities. However, to be truly effective, interventions need to produce lasting improvements in the skills targeted.

Arguably, the best evidenced language intervention is the Nuffield Early Language Intervention (NELI) programme. The NELI programme has now been evaluated in four randomised controlled trials (RCTs; Bowyer‐Crane et al., 2008; Fricke, Bowyer‐Crane, Haley, Hulme, & Snowling, 2013; Fricke et al., 2017; West et al., 2021). These RCTs have demonstrated improvements in language and word reading skills of varying sizes. In addition, one RCT showed that the NELI programme produced improvements in reading comprehension some 6 months after the intervention had finished (Fricke et al., 2013). The largest study of the programme (West et al., 2021) found that children receiving the NELI programme made greater gains in language skills than business‐as‐usual controls (*d* = 0.26 and 0.32 on two different latent variables measuring language ability), as well as small improvements in single word reading. These findings were confirmed by an independent evaluation of the trial conducted by Dimova et al. (2020).

A critical issue, however, is whether the effects of language interventions, such as the NELI programme, lead to durable improvements in language, reading and other skills. A review by Bailey, Duncan, Cunha, Foorman, and Yeager (2020) showed that very few studies evaluating educational interventions included long‐term follow‐up testing, and where they did, there was typically substantial fade out (defined as a reduction in the effect size associated with an intervention). Perhaps most relevant to the current study were the studies of interventions to improve reading skills; for example, Bus and van IJzendoorn's (1999) meta‐analysis of phonological‐awareness training and reading intervention programmes. For studies of phonological awareness training, only seven assessed follow‐up effects which, on average, occurred only 8 months after the end of the programmes. The effect sizes associated with these interventions typically reduced by 50% between the end of treatment and later follow‐up. For studies in which the focus was on reading outcomes, the period of follow‐up was typically longer (averaging 18 months), but there was evidence of very substantial fade out (with the size of the intervention effects typically reducing by roughly 80%). Bailey et al. review a range of other educational interventions, including interventions to increase IQ, executive function and arithmetic skills; in all cases, they report substantial fade out.

Our focus here is on the extent to which the NELI language intervention programme, which has a solid evidence base, leads to sustained improvements in reading and language skills. Previous studies have provided mixed results. For example, Fricke et al. (2013) found substantial improvements in language skills from the NELI intervention (*d* = 0.80) that were maintained at the 6‐month follow‐up (*d* = 0.83). This study also found reliable improvements in phoneme awareness that were maintained 6 months later (*d* = 0.49 at both time points) but no significant improvements in word reading and spelling skills (*d* = 0.14 for a latent variable defined by both measures). In a larger scale RCT, Fricke et al. (2017) found smaller improvements in language skills from the NELI intervention at immediate posttest (*d* = 0.21 after 20 weeks of intervention or *d* = 0.30 after 30 weeks of intervention), but encouragingly these effect sizes were maintained at the 6‐month follow‐up. However, this study found no significant improvements in children's word reading or reading comprehension.

Together these studies suggest that the NELI programme may be associated with relatively durable effects on oral language skills, but smaller and possibly unreliable improvements in word level literacy skills. Only one study of the NELI intervention showed improvements in reading comprehension (Fricke et al., 2013), and in this study, the effects of the intervention on language skills were extremely large (*d* = 0.8).

The durability of intervention effects is an issue of great practical importance. Here, we present data from a long‐term (2‐year‐post‐intervention) follow‐up of children who participated in a large‐scale cluster randomised trial of the NELI intervention (West et al., 2021). This follow‐up was also subject to an independent evaluation commissioned by the Education Endowment Foundation (Groom, Brown, & Lymperis, 2023). We assess the long‐term outcomes on measures of language, single‐word reading and reading comprehension. We tentatively expected to find effects on language skills at long‐term follow‐up but were less sure of any possible long‐term effects on single‐word reading and reading comprehension.

## Method

West et al. (2021) reported data from a cluster‐randomised controlled trial evaluating the effectiveness of the NELI programme that was completed in July 2019. Here, we report data collected at follow‐up in June 2021 approximately 2 years after the original trial was completed.

### Participants

**Figure 1:**
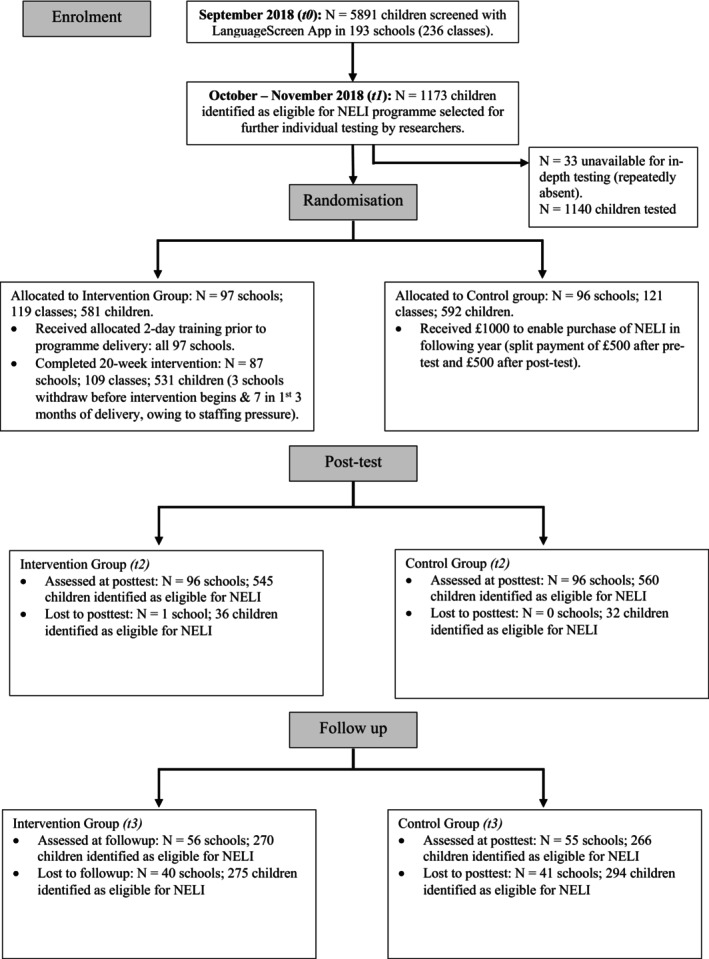
CONSORT diagram showing flow of children through the RCT and follow‐up

Children in the original trial were enrolled on an opt out basis. There were 193 schools in the original trial, with 1,173 children identified as eligible for intervention, 111 schools enrolled in the follow‐up (58%) with 551 children assessed (47% of the sample). Follow‐up assessments (t3) were conducted 23.63 months (*SD* = 0.99 months) after the children had been assessed at posttest (t2). See Figure 1 for the flow of participants through the trial and follow‐up.

### Follow up assessments

The West et al. (2021) trial was completed in July 2019, before the Covid pandemic. The follow‐up assessments reported here took place in the midst of the pandemic (summer 2021). During this period in England, there were frequent school closures and visits to schools were severely restricted to reduce disease transmission. All testing reported here, therefore, had to be done by a nominated member of school staff. Schools were paid to incentivize them to participate. Nominated assessors in each school (typically teaching assistants or teachers) completed online training in how to administer the tests and, if needed, were provided with telephone support by the research team throughout data collection. Assessors uploaded unscored data for all standardised assessments to a secure portal, and scoring was conducted by the research team. All assessment sessions were audio‐recorded by school staff, and a random selection of recordings were compared to the raw data by the research team for quality assurance.

The follow‐up assessments were: (1) *LanguageScreen* (https://oxedandassessment.com/languagescreen/), an automated language assessment used to screen the children in the trial. It includes four subtests (expressive vocabulary, receptive vocabulary, sentence repetition and listening comprehension). Assessments take 10–12 min per child and all scoring is automated; (2) *The Renfrew Action Picture Test* (APT; Renfrew, 2003; information and grammar scores); (3) *YARC Early Word Reading* (Hulme et al., 2009), a measure of single‐word reading; (4) *YARC Passage Reading* – a measure of reading comprehension. To ensure that sufficient questions of suitable difficulty to get an accurate assessment of reading comprehension were administered, each child was asked to read three passages (the Beginner, 1a and 1b texts). After each passage was read, eight comprehension questions were read to the child by the assessor, giving a maximum score of 24 points. Each passage was subject to a discontinuation rule; if a child made 16 or more word reading errors on a passage, they were not administered the comprehension questions.

### The Nuffield Early Language Intervention Programme (NELI)

NELI is a 20‐week programme for children with weak oral language skills delivered by trained teaching assistants. The programme focuses on improving children's vocabulary, developing their narrative skills, encouraging active listening and building confidence in independent speaking. It consists of 3 × 30‐min small group sessions and 2 × 15‐min individual sessions each week (total intervention time: small group sessions 28.5 hr; individual sessions 9.25 hr). It was designed with reference to the Primary Framework for Literacy and Mathematics (DfES, 2006), the Statutory Framework for the Early Years Foundation Stage (DCSF, 2008) and in consultation with teachers and speech and language therapists (Bowyer‐Crane et al., 2008).

### Results

Analyses were conducted in Stata 18.0 (Stata Corp, 2023, College Station, TX, USA). The original trial was pre‐registered (https://doi.org/10.1186/ISRCTN12991126), but the current analyses were not.

Table 1 shows the scores for the control and intervention groups at each test point for all children in the original trial, and separately for the subsample retained at follow‐up, as recommended by Dumville, Torgerson, and Hewitt (2006). Note that the number of items in the LanguageScreen subtests differs across time points, which slightly complicates the interpretation of the values in Table 1. Nevertheless, the general pattern in the means is clear. Children retained at follow‐up (in both the control and intervention group) had better language skills than the total sample at pretest. There are small advantages at follow‐up for the intervention compared to the control group on most language measures, apart from LanguageScreen Receptive Vocabulary and the Action Picture Test information subtests.

**Table 1:** Mean raw scores (*SD*) for intervention and waiting control groups for primary and secondary outcome measures pre‐intervention (t0, t1) and post‐intervention (t2), with effect sizes for intervention effects

	Reliability	Sample included in main trial					Sample retained at follow‐up				
		Intervention		Control		Cohen's *d* [95% CI]	Intervention		Control		Cohen's *d* [95% CI]
		*N*	*M* (*SD*)	*N*	*M* (*SD*)		*N*	*M* (*SD*)	*N*	*M* (*SD*)	
Age (months)											
t1		581	53.22 (3.50)	592	53.40 (3.49)		270	53.20 (3.50)	266	53.16 (3.39)	
LanguageScreen											
Expressive vocabulary											
t1 (24)	.84^a^	563	7.25 (4.16)	585	7.15 (3.92)	0.21 [0.10, 0.32]^b^	264	7.59 (4.09)	264	8.02 (3.79)	0.15 [0.01, 0.30]^c^
t2 (24)		496	11.84 (3.80)	543	10.99 (4.17)		266	12.15 (3.60)	255	11.57 (4.24)	
t3 (24)		–	–	–	–		270	15.72 (3.61)	266	15.64 (3.50)	
Receptive vocabulary											
t1 (31)	.75^a^	581	15.73 (4.15)	591	15.71 (4.16)	0.26 [0.13, 0.39]^b^	270	16.08 (4.13)	265	16.24 (4.16)	0.04 [−0.10, 0.17]^c^
t2 (31)		503	21.40 (3.88)	555	20.36 (4.13)		266	21.79 (3.77)	261	20.74 (4.08)	
t3 (23)		–	–	–	–		270	19.19 (2.99)	266	19.19 (2.59)	
Sentence repetition											
t1 (12)	.87^a^	579	2.47 (2.45)	585	2.44 (2.46)	0.19 [0.05, 0.33]^b^	268	2.50 (2.37)	263	2.59 (2.42)	0.38 [0.19, 0.57]^c^
t2 (12)		503	6.80 (2.83)	550	6.31 (3.27)		266	7.08 (2.80)	259	6.35 (3.33)	
t3 (14)		–	–	–	–		270	10.81 (2.48)	266	10.05 (2.65)	
Listening comprehension											
t1 (12)	.77^a^	580	1.90 (1.99)	585	1.89 (1.90)	0.30 [0.17, 0.43]^b^	269	2.08 (2.03)	266	2.08 (1.90)	0.17 [−0.10, 0.44]^c^
t2 (12)		503	6.12 (2.89)	553	5.30 (2.87)		266	6.36 (2.75)	260	5.37 (3.01)	
t3 (16)		–	–	–	–		270	11.24 (2.88)	266	11.00 (2.81)	
LanguageScreen Total Scores											
t1 (79)	.92^a^	581	27.08 (9.05)	592	27.03 (9.00)	0.32 [0.20, 0.44]^d^	270	28.05 (8.58)	266	28.78 (8.77)	0.23 [0.08, 0.39]^c^
t2 (79)		504	45.91 (10.79)	556	42.58 (11.66)		266	47.39 (9.97)	261	43.69 (11.98)	
t3 (77)		–	–	–	–		270	56.95 (8.61)	266	55.88 (8.25)	
LanguageScreen Standard Scores											
t1		581	84.38 (8.26)	592	84.16 (8.19)		270	85.19 (7.87)	266	85.74 (7.91)	0.23 [0.08, 0.39]^c^
t2		504	103.31 (10.93)	556	87.06 (12.51)		266	104.61 (10.37)	261	88.10 (12.98)	
t3		–	–	–	–		269	100.81 (12.33)	266	98.92 (12.52)	
APT information											
t1 (40)	.86^a^	569	19.19 (7.84)	571	20.09 (7.36)	0.20 [0.09, 0.32]^b^	265	19.94 (7.94)	257	21.15 (7.09)	−0.03 [−0.15, 0.09]^c^
t2 (40)		545	26.24 (5.87)	560	25.38 (6.32)		267	26.59 (5.86)	263	25.94 (6.27)	
t3 (40)		–	–	–	–		269	28.45 (4.77)	263	29.04 (4.42)	
APT grammar											
t1 (38)	.74^a^	569	11.80 (6.86)	571	12.11 (5.57)	0.30 [0.19, 0.41]^b^	265	12.32 (6.85)	257	12.58 (6.37)	0.05 [−0.10, 0.20]^c^
t2 (38)		545	18.89 (6.13)	560	17.25 (6.48)		267	19.15 (5.98)	257	17.54 (6.32)	
t3 (38)		–	–	–	–		269	24.95 (5.44)	263	24.82 (5.57)	
YARC—Early Word Reading											
t1 (30)	.94^e^	569	0.60 (2.97)	571	0.49 (2.38)		265	0.28 (1.52)	257	0.50 (2.17)	0.20 [0.03, 0.37]^c^
t2 (30)		545	8.96 (7.60)	560	8.06 (6.77)		267	9.10 (7.30)	263	7.98 (6.87)	
t3 (30)		–	–	–	–		269	24.91 (5.44)	263	23.79 (8.21)	
YARC—Passage Comprehension											
t3 (24)	.89^f^	–	–	–	–		268	13.70 (6.70)	263	12.89 (6.78)	0.16 [0.00, 0.33]^c^

^a^ Cronbach's alpha calculated at t1.

^b^ Effect size for the intervention based on the difference in progress between groups from the ANCOVA model divided by pooled *SD* for the measure at t1 (see Morris, 2008), robust standard errors are used to correct for clustering within schools.

^c^ Effect size for the intervention based on the difference between groups from Nearest Neighbour Models divided by pooled *SD* for the measure at t1.

^d^ Effect size for the intervention based on the difference in progress between groups from the ANCOVA model in West et al. (2021). Note that the number of items in some of the LanguageScreen subtests differs across times of testing. The total number of items administered at each time point is given in brackets after the time point. LS standard scores at t1 and t2 LS standard scores for the control group are calculated from the LS subtest scores from the 5,879 children screened in the main trial (West et al., 2021); t2 LS standard scores for the intervention group are derived from the t2 control group standard scores; t3 LS standard scores are those created by the LanguageScreen standardisation, which included 348,944 children (Hulme et al., 2024).

^e^ Cronbach's alpha calculated at t2.

^f^ Cronbach's alpha calculated at t3.

### Representativeness of schools contributing follow‐up data

For the pretest (t1) data, we compared the children assessed at follow‐up to those who had dropped out. The two groups did not differ in age (mean age in months: Not Followed Up = 53.42 (*SD* = 3.53); Followed Up = 53.18 (*SD* = 3.45); *t* = 1.20, *p* = .23; *d* = 0.07 [95% CI −0.04, 0.18)] or gender distribution (*χ* ^2^ (1) = 0.20, *p* = .65). However, the language skills at pretest of children followed up were better than the language skills of children not followed up (Mean total LanguageScreen score at pretest: Not Followed Up = 25.91 (*SD* = 9.15); Followed Up = 28.41 (*SD* = 8.67); *t* = 4.80, *p* < .001; *d* = 0.28 [95% CI 0.40, 0.16]). Recruitment to the follow‐up was done at the school level, and schools taking part in the follow‐up were situated in more advantaged postcodes (where language skills would be expected to be better) than those dropping out (mean IDACI decile: Not Followed Up = 4.33 (*SD* = 2.60); Followed Up = 5.31 (*SD* = 2.65); *t* = 6.32; *p* < .001, *d* = 0.37 [95% CI 0.49, 0.26]).

### Differences in patterns of drop‐out between arms

A key issue is whether dropout rates differ between arms in the study, since differential dropout potentially biases any assessment of the effectiveness of the intervention. At follow up, 55 intervention schools provided data on 266 children, and 56 control schools provided data on 270 children. There were very small amounts of missing data at t3, with the total number of children assessed varying between 534 and 536 on the different measures. Most critically, the children in the intervention and control arms were closely matched at pretest on LanguageScreen scores (mean LanguageScreen total raw score: Control 28.78 (*SD* = 8.77); Intervention 28.05 (*SD* = 8.58); *t* = 0.97, *p* = .33; *d* = −0.01 [95% CI −0.120, 0.109)] and age (mean age in months: Control 53.40 (*SD* = 3.49); Intervention 53.22 (*SD* = 3.50); *t* = −0.12, *p* = .91; *d* = 0.05 [95% CI −0.06, 0.16)]. Similarly, there was no significant difference in gender across arms (*χ* ^2^ (1) = 1.05, *p* = .31. In short, there is no evidence of biased attrition between the arms of the study.

In summary, because schools that participated in follow‐up testing had children with better language skills than schools that did not participate, the results reported here may not necessarily generalise to the rest of the sample. We report sensitivity analyses below to address this issue. However, there were no systematic differences at pretest between children in the Control and Intervention arms in terms of language skills or other key variables meaning that the assessment of intervention effects within this subsample should not be biased.

### Statistical tests of the size of intervention effects

The issue at stake here is whether, 2 years after the intervention finished, the NELI intervention has lasting effects on outcomes. Drawing causal inferences here is complicated, given the rate of attrition at follow up, which means that randomisation is no longer intact. Our analyses above suggest that the children retained in the follow‐up do not differ on key characteristics (baseline language skills, age and gender) between the Intervention and Control arms. However, it is still possible that the groups do differ on other (measured or unmeasured) characteristics that may affect response to the intervention. We therefore report analyses that treat these data as quasi‐experimental rather than experimental (since randomisation has been violated due to attrition). We use treatment effect models (nearest‐neighbour matching using a range of relevant measures at pretest (teffects nnmatch in Stata 18) that are more likely to produce unbiased estimates of effect sizes than using ANCOVA models, which assume equivalence on all possible confounders (both measured and unmeasured; see Stuart, 2010). The nearest‐neighbour matching models used here yield an estimate of the average treatment effect (ATE), that is, the difference in mean outcome for those assigned treatment compared to those not assigned treatment. In these models, we used exact matching on dichotomous variables and bias‐adjusted estimators for continuous covariates (as recommended by Abadie & Imbens, 2006, 2011), since we are matching on two or more continuous measures in each model. These are intention‐to‐treat analyses (i.e. they compare outcomes between groups based on group assignment, irrespective of dose of treatment received).

#### LanguageScreen

Our primary outcome measure was the LanguageScreen total score at t3. LanguageScreen is a unidimensional measure that gives a broad and reliable assessment of a child's language skills and has been validated against other measures of language ability (Hulme et al., 2024). The nearest‐neighbour matching model for the LanguageScreen outcome used a range of relevant matching variables at t1: LanguageScreen total score, EAL status, age, gender and Early Word Reading score. The model showed a significant effect of intervention (Average Treatment Effect = 1.89; [95% CI 0.56, 3.21]; *z* = 2.78, *p* = .005; *d* = 0.22).

#### Action picture test

The scores in Table 1 indicate very small advantages for the control group on the APT information and grammar subtests at time 3. The nearest‐neighbour matching model for the t3 APT information outcome used the following matching variables at t1: APT information, EAL status, age, gender and early word reading score. This model confirmed a small nonsignificant effect of intervention (Average Treatment Effect = −0.22; [95% CI −1.12, 0.68]; *z* = −0.49, *p* = .63; *d* = −0.03), as did an equivalent model (with t1 APT grammar in place of t1 APT information as a matching variable) for t3 APT grammar (Average Treatment Effect = 0.31; [95% CI −0.69, 1.30]; *z* = 0.60, *p* = .55; *d* = 0.05).

#### Word reading accuracy (EWR)

At t1, most of the children were essentially non‐readers: 470/537 (88%) of the children could not read a single word on the EWR. Conversely, at time 3 (approximately 2.5 years after t1 testing) children's reading skills had improved considerably: Just 5/535 (1%) could not read a single word, and there was a trend towards a ceiling effect, with 184/535 (34%) children reading all 30 words on the test correctly. The nearest‐neighbour matching model for the t3 Early Word Reading outcome measure used the following matching variables at t1: LanguageScreen total score, EAL status, age, gender and Early Word Reading score. This model showed a non‐significant effect of intervention (Average Treatment Effect = 1.20; [95% CI −0.12, 2.52]; *z* = 1.78, *p* = .07; *d* = 0.16) The effect size here is calculated as the difference in improvement divided by the standard deviation in Early Word Reading at t3, because this measure was at floor at t1. We note that it seems likely that the size of the intervention effect on this measure is downwardly biased because of the ceiling effect operating at follow‐up: 39% of intervention children achieved the maximum score on this test, compared to just 29% of controls.

#### Reading comprehension

Table 1 shows an advantage in reading comprehension scores at time 3 for the Intervention group. The nearest‐neighbour matching model for the t3 Reading Comprehension outcome measure used the following matching variables at t1: LanguageScreen total score, EAL status, age, gender and Early Word Reading. The model revealed a significant effect of intervention (Average Treatment Effect = 1.09; [95% CI 0.00, 2.19]; *z* = 1.96, *p* = .05). As for word reading accuracy, we calculated the effect size here by dividing the average treatment effect by the standard deviation on the measure at follow up (*d* = 0.16). We could not use the standard deviation of the pretest scores (see Morris, 2008), to calculate the effect size because this measure was not administered at pretest.

### Sensitivity analyses

The analyses above show durable improvements from the NELI intervention on LanguageScreen and reading comprehension and a trend towards an effect on word reading accuracy. However, as noted earlier, the schools retained at follow‐up had better LanguageScreen scores than the schools lost to follow‐up. We might expect larger gains from the NELI language intervention in schools with poorer language levels, since children in such schools have more room to improve. To investigate this possibility, we conducted analyses on a subgroup of the followed up schools with lower language levels. For these analyses, we first computed the average LanguageScreen score for each school at pretest. We then selected schools from the follow‐up sample whose mean LanguageScreen raw scores did not differ by more than 0.4 from a school that was lost to follow‐up. This resulted in a reduced sample of 47 control schools and 46 intervention schools containing a total of 519 children. As expected, given the matching, follow‐up schools did not differ at pretest in average language levels from the schools lost to follow‐up (*d* = 0.03). Analyses on this subsample of schools with pupils with lower language levels showed larger effects on each of the key variables than those obtained for the whole sample: LanguageScreen (Average Treatment Effect = 2.58; [95% CI 1.18, 3.99]; *z* = 3.60, *p* = .001; *d* = 0.33), Early Word Reading (Average Treatment Effect = 1.92; [95% CI 0.36, 3.48]; *z* = 2.41, *p* = .016, *d* = 0.22) and Reading Comprehension (Average Treatment Effect = 1.64; [95% CI 0.40, 2.89]; *z* = 2.59, *p* = .01, *d* = 0.24). These analyses indicate that the benefits of the intervention at follow‐up are larger in schools whose children have lower levels of language ability than for the whole sample.

## Discussion

We have reported a long‐term (2‐year) follow‐up study of children who participated in a large cluster‐randomised effectiveness trial of the Nuffield Early Language Intervention (NELI) programme (West et al., 2021). Our primary outcome measure, LanguageScreen, showed relatively large effects of intervention (*d* = 0.22 or *d* = 0.33 for children with lower language ability). In addition, there were benefits on single‐word reading accuracy (*d* = 0.16, or *d* = 0.22 for children with lower language ability) and reading comprehension (*d* = 0.16, or *d* = 0.24 for children with lower language ability). As noted above, because of the effects of the Covid pandemic, we were only able to reassess some 58% of the original sample at follow‐up. This rate of attrition means that we cannot be sure that the effects obtained are truly causal, although the treatment models used here are designed to yield unbiased estimates of causal effects from quasi‐experimental data.

### Interpreting effect sizes

An important issue for this study, and others seeking evidence for the effectiveness of educational interventions, is how to interpret effect sizes. Kraft (2020) reported a meta‐analysis of 1942 effect sizes from 747 RCTs evaluating educational interventions for reading and maths; most interventions produced numerically small effect sizes. Based on this review, Kraft suggested the following benchmarks for interpreting effect sizes (Standardised Mean Differences) in educational interventions: small less than 0.05, medium greater than 0.05 but less than 0.20 and large 0.20 or greater. Based on these benchmarks, the effects reported here, at delayed follow‐up, are medium to large and comparable in size (*d* = 0.16) to that reported in a meta‐analysis of immediate posttest gains from school‐based language interventions (Rogde, Hagen, Melby‐Lervåg, & Lervåg, 2019).

Evaluating the practical significance of effect sizes from interventions is a complex issue. As Kraft notes, when defending his benchmark of .20 as a large effect size, ‘raising student achievement by 0.20 *SD* results in a 2% increase in annual lifetime earnings on average’. Two other factors that Kraft emphasises are critical for evaluating the educational importance of effect sizes are cost and scalability – because interventions that are inexpensive and scalable may be important even if their associated effect sizes are small. This aligns with arguments from public health that small effect sizes may be of considerable importance. Carey, Ridler, Ford, and Stringaris (2023) report simulations showing that an increase of *d* = 0.20 on a measure of adolescent mental health would be expected to result in an increase in referrals for depression of almost 250,000 cases in a population of 10 million (and a tiny effect of *d* = 0.05, would result in an increase of over 50,000 referrals).

The benefits demonstrated here for the NELI intervention therefore need to be interpreted in relation to Kraft's criteria of cost and scalability. According to the Education Endowment Foundation the NELI programme's cost is ‘very low’. Furthermore, there is evidence that NELI works at scale (Smith, Staunton, Sahasranaman, & Worth, 2023) producing similar effect sizes in a national rollout to over 10,000 primary schools in England to those found in the RCT reported by West et al. (2021). In summary, the effect sizes reported here, from a large‐scale follow‐up study, can be seen as medium to large, and in the light of the NELI programme's low cost and scalability, are deemed to be educationally important.

### The effects of language intervention on reading comprehension

The effects of the NELI programme on reading comprehension were significant and of an educationally significant size. It is probably relevant that the only previous study to find transfer effects from the NELI programme to reading comprehension reported an extremely large effect on language outcomes (Fricke et al., 2013). The current study is important in providing further evidence that language intervention, in isolation from any work on reading, may benefit reading comprehension (see also Clarke et al., 2010). Findings of transfer from language interventions to reading comprehension are rare, however. A meta‐analysis by Elleman, Lindo, Morphy, and Compton (2009) showed no significant transfer from isolated vocabulary instruction to reading comprehension, which is perhaps not surprising. The meta‐analysis by Rogde et al. (2019) identified 16 studies that had examined transfer from linguistic comprehension interventions to reading comprehension. They found overall negligible effects. However, 9/16 of these studies focused exclusively on vocabulary instruction rather than broader language skills including narrative and grammatical skills. We believe that evidence from this study and others targeting broader oral language skills suggests that broad‐based language interventions can benefit reading comprehension. Further work is clearly needed to establish the robustness and moderators of such effects. We would emphasise that we can only expect transfer from language interventions to reading comprehension when such studies produce meaningful improvements in oral language skills.

### The mechanisms underlying improvements in language skills

At delayed follow‐up, the NELI programme produces improvements in language and reading skills, with the strongest effect on a broad‐based measure of receptive and expressive language (LanguageScreen). The fact that these effects persist 2 years after the intervention was completed has important educational implications since such effects are likely to contribute to improvements in many aspects of education and well‐being. Theoretically, more work is needed to understand the factors that account for these longer term effects. The NELI programme consists of direct training of key language skills (vocabulary, narrative production and listening skills), as well as training in meta‐cognitive strategies (stressing that children need to attend, only speak at appropriate times and ask when they do not understand something). We speculate that the long‐term effects on language obtained here reflect some enduring meta‐cognitive changes in children who have received the programme. As well as learning specific information (the meanings of words taught in the programme), the children have learned that language is a system that can be mastered, and we believe the strategies they have been taught (e.g. being attentive and listening carefully; asking about the meanings of words that they do not understand; using context to help understand words that are unfamiliar) may have enduring effects on their language learning ability. One particularly important feature of the NELI programme is that children are encouraged to speak, with structured support used by the Teaching Assistants to scaffold and expand their utterances. There is evidence that language production may be particularly critical in language learning (MacDonald, 2013).

### Interpreting fade out

Fade out refers to a reduction in the effect size associated with an intervention as time progresses. The current study indicates that the NELI intervention shows a relatively small degree of fade out (the effect size here for the whole sample on LanguageScreen (*d* = 0.22) is smaller than that found in the original trial (*d* = 0.33) but still substantial).

Fade out might arise because intervention children show declines, or because control children catch up. The LanguageScreen raw scores in Table 1 show that both the intervention and control groups increase their scores between the end of the intervention and follow‐up testing, but the degree of increase is larger in the control group. This pattern might be partially a statistical effect of regression to the mean (given that control children start out with lower language scores than the controls, regression to the mean would be expected to give larger improvements in this group). However, it is also possible that control schools, which were made aware of the language difficulties experienced by some of their children, provided extra support to ameliorate such difficulties.

### Limitations

This study reports a long‐term follow‐up of children who participated in an effectiveness trial of the NELI programme. A limitation of this study is the high rate of attrition, which means that care must be taken in making causal claims. However, the analyses reported are designed to minimise bias and provide accurate estimates of causal effects, where such effects exist. Nevertheless, further studies in which long‐term follow‐up is built into the design are needed to support the current findings. The practical challenges encountered here, including the fact that all testing had to be conducted by school staff due to the schools being closed to visitors during the Covid pandemic, were considerable. In practice, it may be difficult to obtain long‐term follow‐up data in educational trials such as this without encountering appreciable attrition. Nevertheless, this is an important challenge for future studies to address.

## Ethical considerations

The study received ethical approval from the Department of Education Ethics Committee at The University of Oxford (reference: ED‐C1A‐19‐239). The approval covered the initial trial and permission to follow‐up participants. Schools (usually the head teacher) gave informed consent and communicated details of the proposed research‐based intervention. Parents were asked to opt out should they not wish for their child to participate.

**Key points**

- Oral language skills are critical for education and psychosocial development.
- Follow‐up data from a randomized controlled trial show that a 20‐week language intervention produces sustained improvements in language and reading skills.
- Delayed follow‐ups in educational interventions are rare and are typically associated with substantial fade‐out effects; the current findings are important in providing evidence of enduring effects.
- These findings have important implications for educational policy and suggest that language interventions may be one way of helping to reduce social inequalities in educational and mental health outcomes.

## Data Availability

Data are available from the corresponding author (CH).

## References

[jcpp14157-bib-0001] Abadie, A. , & Imbens, G.W. (2006). Large sample properties of matching estimators for average treatment effects. Econometrica, 74, 235–267.

[jcpp14157-bib-0002] Abadie, A. , & Imbens, G.W. (2011). Bias‐corrected matching estimators for average treatment effects. Journal of Business & Economic Statistics, 29, 1–11.

[jcpp14157-bib-0003] Bailey, D.H. , Duncan, G.J. , Cunha, F. , Foorman, B.R. , & Yeager, D.S. (2020). Persistence and fade‐out of educational‐intervention effects: Mechanisms and potential solutions. Psychological Science in the Public Interest, 21, 55–97. 33414687 10.1177/1529100620915848 PMC7787577

[jcpp14157-bib-0004] Bowyer‐Crane, C. , Snowling, M.J. , Duff, F.J. , Fieldsend, E. , Carroll, J. , Miles, J.N.V. , … & Hulme, C. (2008). Improving early language and literacy skills: Differential effects of an oral language versus a phonology with reading intervention. Journal of Child Psychology & Psychiatry, 49, 422–432. 18081756 10.1111/j.1469-7610.2007.01849.x

[jcpp14157-bib-0006] Bus, A.G. , & Van IJzendoorn, M.H. (1999). Phonological awareness and early reading: A meta‐analysis of experimental training studies. Journal of Educational Psychology, 91, 403.

[jcpp14157-bib-0007] Carey, E.G. , Ridler, I. , Ford, T.J. , & Stringaris, A. (2023). Editorial Perspective: When is a ‘small effect’ actually large and impactful? Journal of Child Psychology and Psychiatry, 64, 1643–1647. 37226639 10.1111/jcpp.13817

[jcpp14157-bib-0008] Chow, J.C. , & Ekholm, E. (2019). Language domains differentially predict mathematics performance in young children. Early Childhood Research Quarterly, 46, 179–186.

[jcpp14157-bib-0040] Clarke, P. , Snowling, M. , Truelove, E. , & Hulme, C. (2010). Ameliorating children's reading comprehension difficulties: A randomised controlled trial. Psychological Science, 21, 1106–1116. 20585051 10.1177/0956797610375449

[jcpp14157-bib-0009] DCSF . (2008). Statutory Framework for the Early Years Foundation Stage. Nottingham: Department for Children, Schools and Families.

[jcpp14157-bib-0010] DfES . (2006). Primary Framework for Literacy and Mathematics. Norwich: Department for Education and Skills.

[jcpp14157-bib-0011] Dimova, S. , Ilie, S. , Brown, E.R. , Broeks, M. , Culora, A. , & Sutherland, A. (2020). The Nuffield Early Language Intervention. London: Education Endowment Foundation.

[jcpp14157-bib-0012] Dumville, J.C. , Torgerson, D.J. , & Hewitt, C.E. (2006). Reporting attrition in randomised controlled trials. British Medical Journal, 332, 969–971. 16627519 10.1136/bmj.332.7547.969 PMC1444839

[jcpp14157-bib-0013] Elleman, A.M. , Lindo, E.J. , Morphy, P. , & Compton, D.L. (2009). The impact of vocabulary instruction on passage‐level comprehension of school‐age children: A meta‐analysis. Journal of Research on Educational Effectiveness, 2, 1–44.

[jcpp14157-bib-0014] Fricke, S. , Bowyer‐Crane, C. , Haley, A.J. , Hulme, C. , & Snowling, M.J. (2013). Efficacy of language intervention in the early years. Journal of Child Psychology and Psychiatry, 54, 280–290. 23176547 10.1111/jcpp.12010 PMC3593174

[jcpp14157-bib-0015] Fricke, S. , Burgoyne, K. , Bowyer‐Crane, C. , Kyriacou, M. , Zosimidou, A. , Maxwell, L. , & Hulme, C. (2017). The efficacy of early language intervention in mainstream school settings: A randomized controlled trial. Journal of Child Psychology and Psychiatry, 58, 1141–1151. 28524257 10.1111/jcpp.12737

[jcpp14157-bib-0016] Groom, M. , Brown, R.B. , & Lymperis, L. (2023). The Nuffield Early Language Intervention addendum report. London: Education Endowment Foundation.

[jcpp14157-bib-0017] Guo, G. , & Harris, K.M. (2000). The mechanisms mediating the effects of poverty on children's intellectual development. Demography, 37, 431–447. 11086569 10.1353/dem.2000.0005

[jcpp14157-bib-0018] Hart, B. , & Risley, T.R. (1995). Meaningful differences in the everyday experience of young American children. Baltimore, MD: Paul H Brookes.

[jcpp14157-bib-0019] Hjetland, H.N. , Brinchmann, E.I. , Scherer, R. , Hulme, C. , & Melby‐Lervag, M. (2020). Preschool pathways to reading comprehension: A systematic meta‐analytic review. Educational Research Review, 30, 100323.

[jcpp14157-bib-0021] Hornburg, C.B. , Schmitt, S.A. , & Purpura, D.J. (2018). Relations between preschoolers' mathematical language understanding and specific numeracy skills. Journal of Experimental Child Psychology, 176, 84–100. 30145520 10.1016/j.jecp.2018.07.005

[jcpp14157-bib-0022] Hulme, C. , McGrane, J. , Duta, M. , West, G. , Cripps, D. , Dasgupta, A. , … & Snowling, M. (2024). LanguageScreen: The development, validation, and standardization of an automated language assessment app. Language, Speech, and Hearing Services in Schools, 55, 904–917. 38776269 10.1044/2024_LSHSS-24-00004

[jcpp14157-bib-0023] Hulme, C. , Nash, H.M. , Gooch, D. , Lervag, A. , & Snowling, M.J. (2015). The foundations of literacy development in children at familial risk of dyslexia. Psychological Science, 26, 1877–1886. 26525072 10.1177/0956797615603702 PMC4676358

[jcpp14157-bib-0025] Hulme, C. , Stothard, S.E. , Clarke, P. , Bowyer‐Crane, C. , Harrington, A. , Truelove, E. , & Snowling, M.J. (2009). York assessment of reading for comprehension: Early reading [Measurement instrument]. London: GL Assessment.

[jcpp14157-bib-0026] Kraft, M.A. (2020). Interpreting effect sizes of education interventions. Educational Researcher, 49, 241–253.

[jcpp14157-bib-0027] MacDonald, M.C. (2013). How language production shapes language form and comprehension. Frontiers in Psychology, 4, 226. 23637689 10.3389/fpsyg.2013.00226 PMC3636467

[jcpp14157-bib-0028] Morris, S.B. (2008). Estimating effect sizes from pretest‐posttest‐control group designs. Organizational Research Methods, 11, 364–386.

[jcpp14157-bib-0030] Renfrew, C. (2003). Action picture test. Milton Keynes, UK: Speechmark.

[jcpp14157-bib-0031] Rogde, K. , Hagen, Å.M. , Melby‐Lervåg, M. , & Lervåg, A. (2019). The effect of linguistic comprehension instruction on generalized language and reading comprehension skills: A systematic review. Campbell Systematic Reviews, 15, e1059. 37131857 10.1002/cl2.1059 PMC8356536

[jcpp14157-bib-0032] Roulstone, S. , Law, J. , Rush, R. , Clegg, J. , & Peters, T. (2011). Investigating the role of language in children's early educational outcomes. Available from: https://assets.publishing.service.gov.uk/government/uploads/system/uploads/attachment_data/file/181549/DFE‐RR134.pdf

[jcpp14157-bib-0033] Sampson, R.J. , Sharkey, P. , & Raudenbush, S.W. (2008). Durable effects of concentrated disadvantage on verbal ability among African–American children. Proceedings of the National Academy of Sciences of the United States of America, 105, 845–852. 18093915 10.1073/pnas.0710189104 PMC2242679

[jcpp14157-bib-0041] Sirin, S.R. (2005). Socioeconomic status and academic achievement: A meta‐analytic review of research. Review of Educational Research, 75, 417–453.

[jcpp14157-bib-0034] Smith, A. , Staunton, R. , Sahasranaman, A. , & Worth, J. (2023). Impact Evaluation of Nuffield Early Language Intervention (NELI) Wave Two. London: Education Endowment Foundation.

[jcpp14157-bib-0035] Snow, P.C. (2016). Elizabeth Usher Memorial Lecture: Language is literacy is language‐positioning speech‐language pathology in education policy, practice, paradigms and polemics. International Journal of Speech‐Language Pathology, 18, 216–228. 27063684 10.3109/17549507.2015.1112837 PMC4906364

[jcpp14157-bib-0037] StataCorp . (2023). Stata statistical software: Release 18. College Station, TX: StataCorp LLC.

[jcpp14157-bib-0038] Stuart, E.A. (2010). Matching methods for causal inference: A review and a look forward. Statistical Science, 25, 1–21. 20871802 10.1214/09-STS313 PMC2943670

[jcpp14157-bib-0039] West, G. , Snowling, M.J. , Lervag, A. , Buchanan‐Worster, E. , Duta, M. , Hall, A. , … & Hulme, C. (2021). Early language screening and intervention can be delivered successfully at scale: Evidence from a cluster randomized controlled trial. Journal of Child Psychology and Psychiatry, 62, 1425–1434. 33783013 10.1111/jcpp.13415

